# Design and performances of a compact differential pumping unit for X-ray beamlines

**DOI:** 10.1107/S1600577525007945

**Published:** 2025-10-10

**Authors:** Cristian Maccarrone, Anne-Lise Buisson, Marco Cammarata

**Affiliations:** a ESRF – The European Synchrotron, 71 Avenue des Martyrs, 38000Grenoble, France; University of Malaga, Spain

**Keywords:** differential pumping unit, windowless setup, X-ray beamlines, turbopump

## Abstract

In this contribution, we present a compact differential pumping unit with apertures ≥500 µm. It allows windowless operation for in-air sample environments as well as to connect low-quality in-vacuum sample environments to the beamline UHV (≤10^−8^ mbar) section. The unit also protects the UHV section of the beamline from accidental venting due to operator errors. To simplify the design, an adjustment-free series of pinholes is used. The positioning of the apertures relies on tight machining tolerances. The assembly consists of just eight parts: one main aluminium body, four threaded cylinders with apertures and three covers with links to pumping units assembled with Viton seals. The overall footprint is restricted to 368 mm on the beam axis.

## Abstract 2

We present a compact differential pumping unit with apertures ≥500 µm. It allows windowless operation for in-air sample environments as well as to connect low-quality in-vacuum sample environments to the beamline UHV section. The unit also protects the UHV section from accidental venting. The overall footprint is restricted to 368 mm on the beam axis.

## 1. Introduction

Many modern X-ray experiments rely on high-intensity (≥10^12^ photons s^−1^), tightly focused (≤30 µm) X-ray beams. The use of X-ray windows is also very common to separate the sample area from the high or ultra-high vacuum sections. X-ray windows are often made of thin polymeric (Kapton, Mylar, *etc.*) or inorganic (Si_3_N_4_, diamond *etc.*) materials. When X-ray beams pass through windows, a small, but often measurable, fraction of radiation is scattered, producing unwanted background. While, at higher scattering angles, slits can be used to shield such unwanted contribution, this becomes increasingly difficult at smaller angles and impossible in forward scattering geometry.

Moreover, windows are known to become ‘dirty’ over time. While deposition of organic matter can be mitigated using a stream of clean gas, carbon deposits can be generated by the interaction of the intense X-ray beam with residual gases typically present in vacuum systems. This is more relevant for *in situ*/*operando* experiments when gases are intentionally introduced into the sample environment.

The above-mentioned effects thus result in a higher than expected, and often time-dependent, background scattering signal that can be detrimental for studying weakly scattering samples.

Lastly, windows may distort the wavefront of the X-ray beam, thus deteriorating the experiments that exploit the improved coherence of fourth-generation X-ray sources (Raimondi *et al.*, 2023 ▸).

For these reasons, several experiments would benefit from windowless operation. Unfortunately, even when using in-vacuum sample environments, this is far from trivial as the vacuum level at the sample is typically much worse than the ‘beamline vacuum’ (typically in the UHV range). In such cases, a gate valve (to be closed upon sample changes and re-opened before data collection) is of no use. Moreover, the use of gate valves is prone to operator mistakes that might result in the venting of a section of the beamline.

To greatly simplify the use of windowless operation, differential pumping units (DPUs) can be used. They consist of two or more chambers separated by small apertures. Each chamber is pumped via a pumping port. This staged approach allows us to connect sections that have different pressures together, progressively lowering the pressure. Note that DPUs are not new. They have played a key role in the development of X-ray photoelectron spectroscopy (Siegbahn, 1981 ▸) and have been developed and used at almost all large scale facilities (Giuliani *et al.*, 2009 ▸; Pérez-Dieste *et al.*, 2013 ▸; Gog *et al.*, 2007 ▸; Renier & Draperi, 1997 ▸).

**Figure 1:**
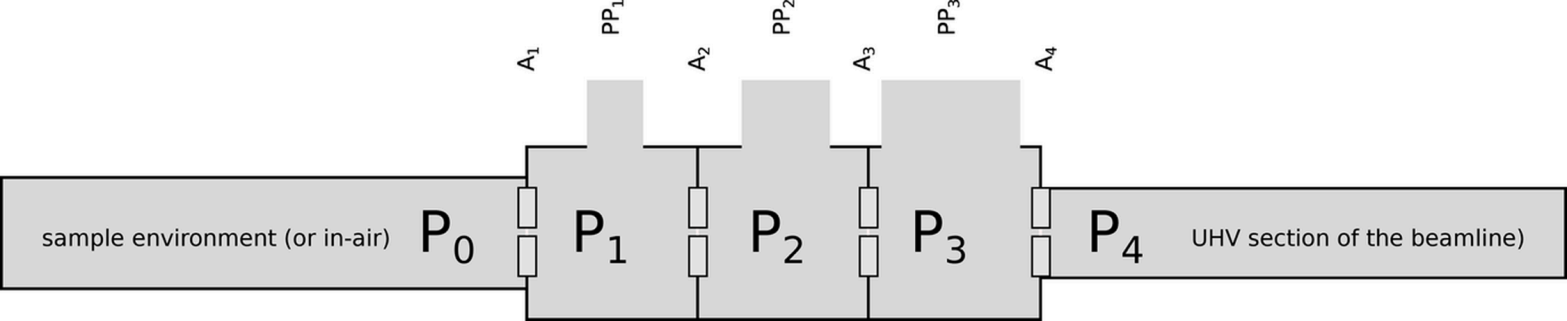
Schematic configuration of the differential pumping scheme adopted. Three stages (1 to 3) are separated by small (<1 mm) apertures (indicated as A_1_–A_4_). P_0_ indicates the pressure at the sample (that can be equal to the ambient pressure). P_4_ is the required vacuum level of the beamline (typically 10^−8^ mbar). PP_*x*_ indicates the pumping ports.

Our development, similar to the work of Tamenori (2010 ▸), is sketched in Fig. 1 ▸; *P*_0_ is the pressure present in the sample environment chamber. *P*_4_ is the final pressure that should be reached. We aimed to achieve:

(i) The possibility to work with an entrance pressure (*P*_0_) up to atmospheric pressure.

(ii) Operation in the UHV section [*P*_4_ in the low 10^−8^ mbar range when using a small (∼55 l s^−1^) ion pump].

(iii) Limited footprint allowing installation in beamlines already designed and operational.

(iv) A small number of pumps used to minimize cost and required space at the beamline.

(v) Limit vibrations as much as possible, in particular at low frequencies as these are more difficult to damp.

(vi) Relatively large apertures, first aperture of 0.2 (or 0.5) mm diameter, all other apertures should be in the 0.5 mm to 1 mm range.

The resulting system has a footprint of only 368 mm, uses two pumps (one scroll and one turbomolecular), and has been used in operation at the ID10-COH endstation of ESRF since June 2024.

## 2. Vacuum considerations

This section does not aim to provide an in-depth discussion on how the expected vacuum levels can be calculated. The interested reader can find a more in-depth approach elsewhere (*e.g.* Tamenori, 2010 ▸).

The idea was to use a three-stage approach. The first chamber (which is connected to atmospheric pressure through the first aperture) is pumped by a primary pump via a DN-40 port. The primary pump can by positioned outside the experimental hutch to reduce vibration and noise. The first aperture (A_1_) determines the maximum air-flow the system should be capable to cope with.

The second and third sections are pumped via a single turbopump with side port pumping (Agilent TwisTorr 305 SF). The side port (KF-40), providing a pumping speed of about 8 l s^−1^ for nitrogen, is used to pump the second section via PP_2_ (see Fig. 1 ▸); the third section (PP_3_) is pumped by the main inlet of the turbopump.

The choice to use a single turbopump was driven by the need to have a compact and cost-effective system as well as to avoid low-frequency vibrations generated by the ‘beating’ vibrations (due to the slightly different pumps’ speed). These beat vibrations can have quite low frequencies and high magnitudes making their damping difficult or even impossible.

When dimensioning the system, it is crucial to respect the pump’s maximum compression ratio between the fore-side-inlet ports. Failure to do so may limit the pressure in the P_3_ chamber due to gas back-streaming from the fore-line or side-port.

The apertures’ conductance values have been estimated by combining well known formulae from the literature (Roth, 1990 ▸; Lafferty, 1998 ▸) as well as commercial software (*Molflow* and *VacTran*).

### 2.1. Dimensioning and laboratory test

To validate the vacuum design, laboratory tests have been carried out with the following configuration:

(i) A_1_ = 0.2 mm, A_2_ = 0.5 mm and A_3_ = 1 mm; A_4_ = 1 mm in calculations (closed in the test).

(ii) 15 m^3^ h^−1^ primary pump via 2 m-long, 10 mm inner-diameter rubber tube on PP_1_, resulting in about 3 l s^−1^ effective pumping speed (used also on the fore-line of the turbopump).

(iii) 4 l s^−1^ on PP_2_ by the side port of turbopump (considering conductance limitations).

(iv) 200 l s^−1^ on PP_3_ by the main inlet of turbopump (considering conductance limitations).

(v) A 55 l s^−1^ ion pump in the UHV section (*P*_4_).

In Table 1 ▸, calculated and measured pressures are reported. Note that measures have been taken without the ion pump on the *P*_4_ chamber. The last aperture (A_4_) was closed during the test, but this did not affect the pressure in the chambers pumped by primary and turbo, which were the goal of the test.

The pressure ratio between the side-port (*P*_2_) and fore-line (*P*_1_) is about 100, below the limit of the turbopump that is 200 for nitrogen. For the main inlet (*P*_3_) and fore-line (*P*_1_), the measured ratio is 5 × 10^5^, again well below the limit of the pump that is 10^8^. Last, but not least, the pressure ratio between the main inlet (*P*_3_) and the side-port (*P*_2_) is 5 × 10^3^, that is still below the limit of 10^5^ of the pump.

The turbopump’s power was estimated using iso-flow/iso-power curves and confirmed by measurements. Low power is crucial to avoid air or water cooling, which can cause vibrations. The resulting value of 33 W, primarily caused by fore-line pressure, is low enough to operate the pump without cooling (the temperature reported by the turbopump controller was 38°C).

### 2.2. Larger A_1_ aperture with dedicated primary pump for the turbopump fore-line

The proposed solution offers some margins to increase the smallest aperture diameter. In particular, A_1_ can be increased by increasing the available pumping speed at *P*_1_. For instance, with A_1_ = 0.5 mm and the first chamber pumped by a 40 m^3^ h^−1^ primary with a 4 m-long, 25 mm inner-diameter rubber tube, we can estimate a *P*_1_ of 3.6 mbar with basically no variation on the other pressures (*P*_2_ to *P*_4_). In this case, the power of the turbopump will increase due to higher fore-line pressure. The use of a smaller, separate primary pump could be envisaged to avoid the need of active cooling of the turbopump. This is the current configuration used for the installation at the ID10-COH beamline (in Table 2 ▸, estimated and measured pressures). The second primary pump was already present to sustain another turbopump on an adjacent section, and the fore-line pressure was in the 10^−1^ mbar, resulting in even lower power on the turbopump compared with the laboratory test (Table 1 ▸).

Note that by pumping air via the first aperture, a significant amount of H_2_O reaches the first scroll pump with possible condensation and corrosion over a long time. We thus foresee purging with the ballast open for one night every ∼3 months.

## 3. Mechanical considerations

**Figure 2:**
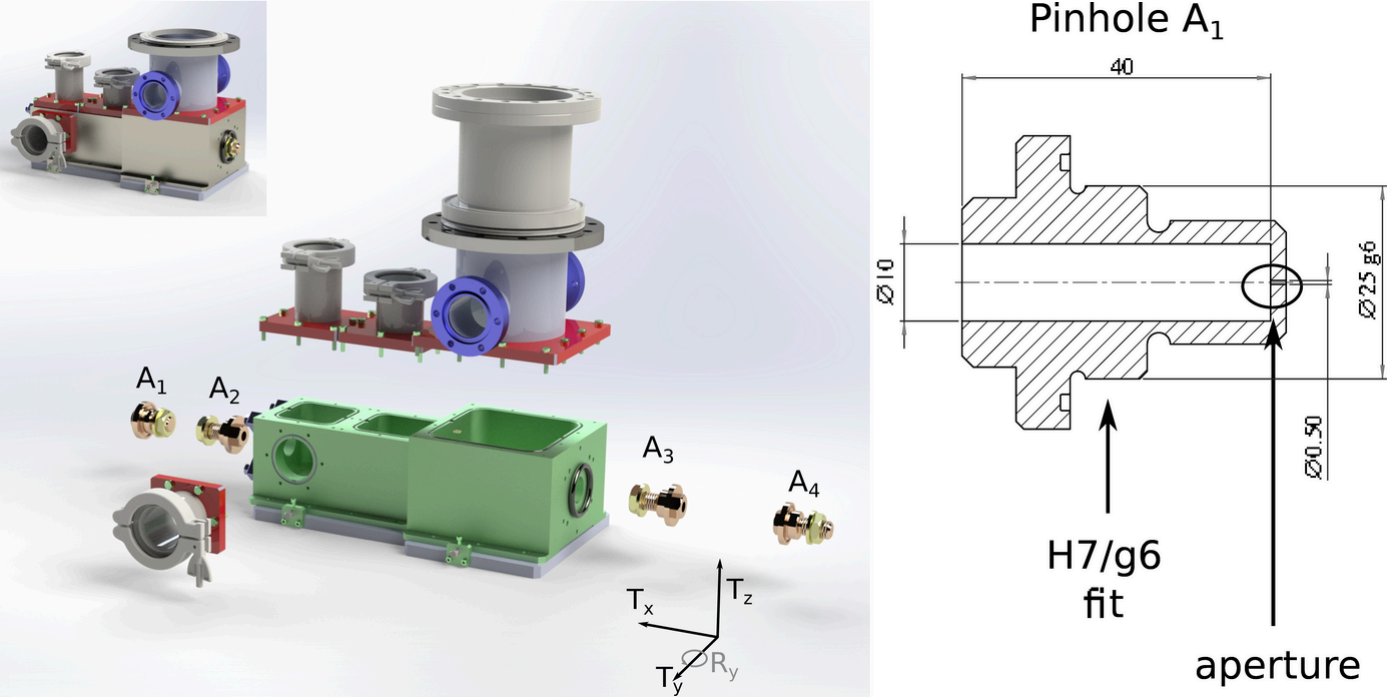
Mechanical assembly. (Left) 3D rendering along with an exploded view of the main components. (Right) Cross section of the A_1_ aperture with the reference surface and the aperture highlighted. The coordinate system is also shown. The beam propagates along *x*. *T*_*x*,*y*,*z*_ indicate the translations and *R*_*x*,*y*,*z*_ the rotations (only *R*_*y*_ is shown for clarity).

As mentioned earlier, the goal was to design a very compact system. This implies the need to avoid any adjustment of the aperture position and relying on tight machining tolerances. Fig. 2 ▸ shows the main components.

The main aluminium body includes three vacuum chambers and a central hole, which is machined with a 20 µm parallelism tolerance relative to two external reference surfaces. Each small aperture (200 µm, 500 µm and 1 mm in diameter) was drilled into a separate stainless steel cylindrical insert, with a coaxiality tolerance of ±5 µm between the aperture and the reference cylinder. The final assembly is based on an H7/g6 fit, allowing ±20 µm clearance between the central hole in the aluminium body and each aperture insert.

**Figure 3:**
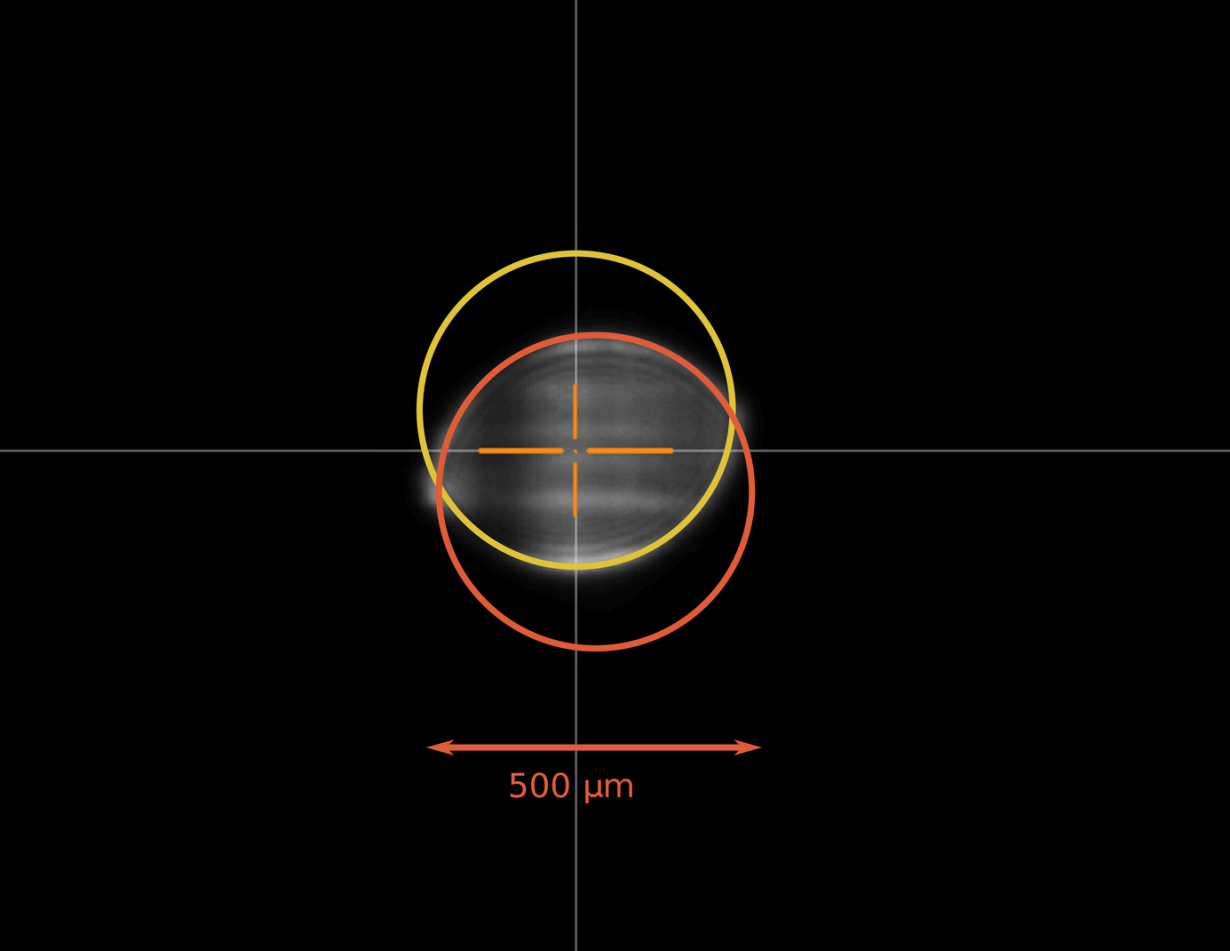
Radiography recorded using a high-resolution objective coupled to a fluorescent X-ray screen (YAG:Ce). A collimated, large (700 µm FWHM) attenuated beam was sent through the DPU in its working position (last aperture ∼25 cm upstream of the sample position). The high-resolution screen was positioned ∼7 m after the sample position. For perfectly aligned apertures, a perfect circular shadow is expected. The interferences due to the highly coherent beam are visible. The two circles have a diameter of 500 µm. The distance between the two centres, indicative of the machining tolerances, is 133 µm.

**Figure 4:**
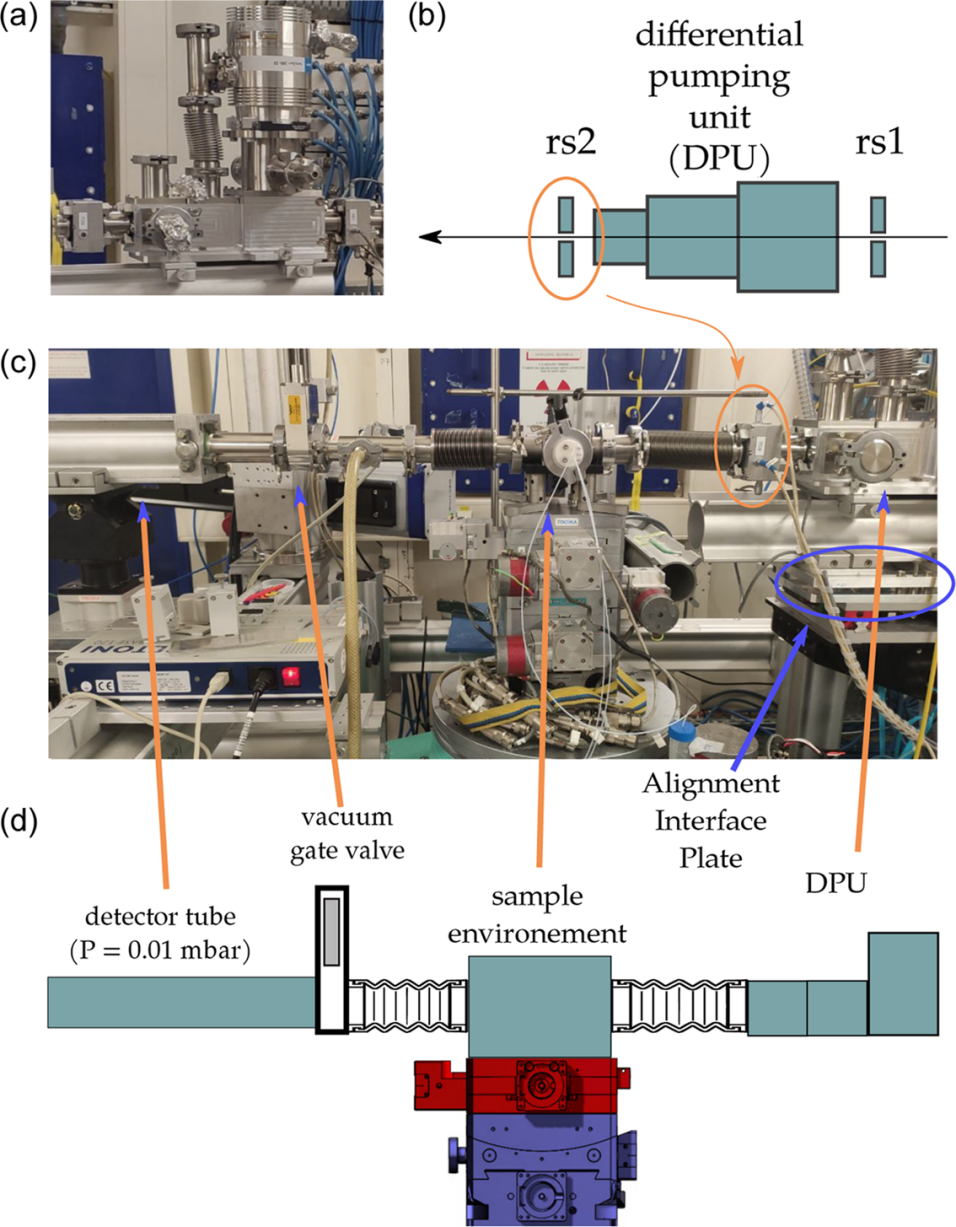
(*a*) Photograph of the DPU installed at ID10-COH. (*b*) Schematic of the DPU setup showing in particular the two sets of slits (installed upstream and downstream). The upstream slit is sometimes used to define the beam, the downstream slit is used as a clean-up slit. (*c*) Photograph of the DPU as integrated with an (in-vacuum) sample environment. Highlighted are one of the two sets of slits (rs2) as well as the sample environment and gate valve. The gate valve is used to isolate the detector tube while replacing the sample. (*d*) Schematic illustrating the DPU configuration used for several experiments: the sample environment is connected to the DPU via a flexible connection (KF16 bellow) and to the ‘detector tube’ (typically 4 to 7 m long). For replacing the sample, the gate valve is closed, the sample chamber vented. Once the sample is replaced, the sample chamber is pumped down before the gate valve is opened.

On the beamline, the unit is aligned using a combination of translations and rotations. The coordinate system is shown in Fig. 2 ▸. Translation along the beam propagation direction (*x*) and rotation around this axis are not critical. Perpendicular translations (*T*_*y*_ and *T*_*z*_) are motorized and used quite regularly. Rotation around these two axes is, in principle, possible using the positioning system of the granite table supporting the DPU and several other components (shutters, filters *etc.*); for this reason only small angular adjustments (∼1–2 mrad) are possible without using the alignment interface plate shown in Fig. 4(*c*). The unit was pre-aligned by the ESRF alignment group and is routinely tweaked using the X-ray beam and a photodiode and/or high-resolution screen. Considering that the last two apertures are the smallest, they will define the beam axis. We can estimate the maximum cumulative machining errors to less than 70 µm [*i.e.* 20 µm (parallelism) + 2 × 20 µm (H7/g6 play) + 2 × 5 µm coaxiality tolerance = 70 µm]. However, the ‘radiography’ measurement, using a large collimated beam and an high-resolution camera, revealed a misalignment of 133 µm (see Fig. 3 ▸). A metrology inspection identified the faulty component as the 500 µm (A_1_) aperture, showing a coaxiality error of 100 µm between the drilled aperture hole and its theoretical position. Corrective action will involve re-machining the aperture to meet the ±5 µm coaxiality tolerance. For the time being, all the results presented have been obtained with the defective component, pending the machining of the new part.

## 4. Performances and use at the beamline

The DPU has been installed at the ID10-COH endstation of ESRF in June 2024 and used for all experiments since its installation. In this section, we will discuss the beamline integration and use.

ID10-COH exploits the highly coherent X-ray beam produced by the upgraded ESRF storage ring (ESRF-EBS) (Raimondi *et al.*, 2023 ▸) to study structure and dynamics by coherent X-ray scattering. Today, thanks to the high flux, dynamics down to sub-microsecond (Chushkin *et al.*, 2025 ▸) as well as sub-10 nm resolution in 3D (Chushkin & Zontone, 2025 ▸) can be achieved. A good fraction of the experiments are performed in forward scattering where working windowless is particularly useful.

The unit mounted in the experimental hutch is shown in Fig. 4 ▸. Two sets of slits (one upstream and one downstream) are connected to the DPU via KF40 flanges and o-rings. The turbomolecular pump, pumping stage 2 via its side port and stage 3 via its main flange, is also clearly visible. Typical values for the vacuum level in each section and turbomolecular pump consumption are similar to the values reported in Table 2 ▸ when the chamber is not connected to an in-vacuuum sample environment.

As mentioned above, two primary pumps are used. The first (Pfeiffer ACP 40) is used to pump stage 1 via 25 mm-diameter, 4 m-long tube. The second pump (Pfeiffer ACP 15) is used to pump the fore-line flanges of all turbomolecular pumps in the experimental hutch. In principle, the pump used for stage 1 can be used to pump the turbomolecular fore-line but at the cost of increased load when using with in-air setups.

To limit the number of pumps needed, we have initially used the DPU with an entrance aperture (A_1_) of 200 µm. Unfortunately, when using beams with an FWHM size of ∼30 µm (or larger), the beam’s tails as well as the diffraction from rs1 would touch A_1_ and produce ‘flares’ in forward scattering (up to 1–3 mrad of scattering angle).^1^

**Figure 5:**
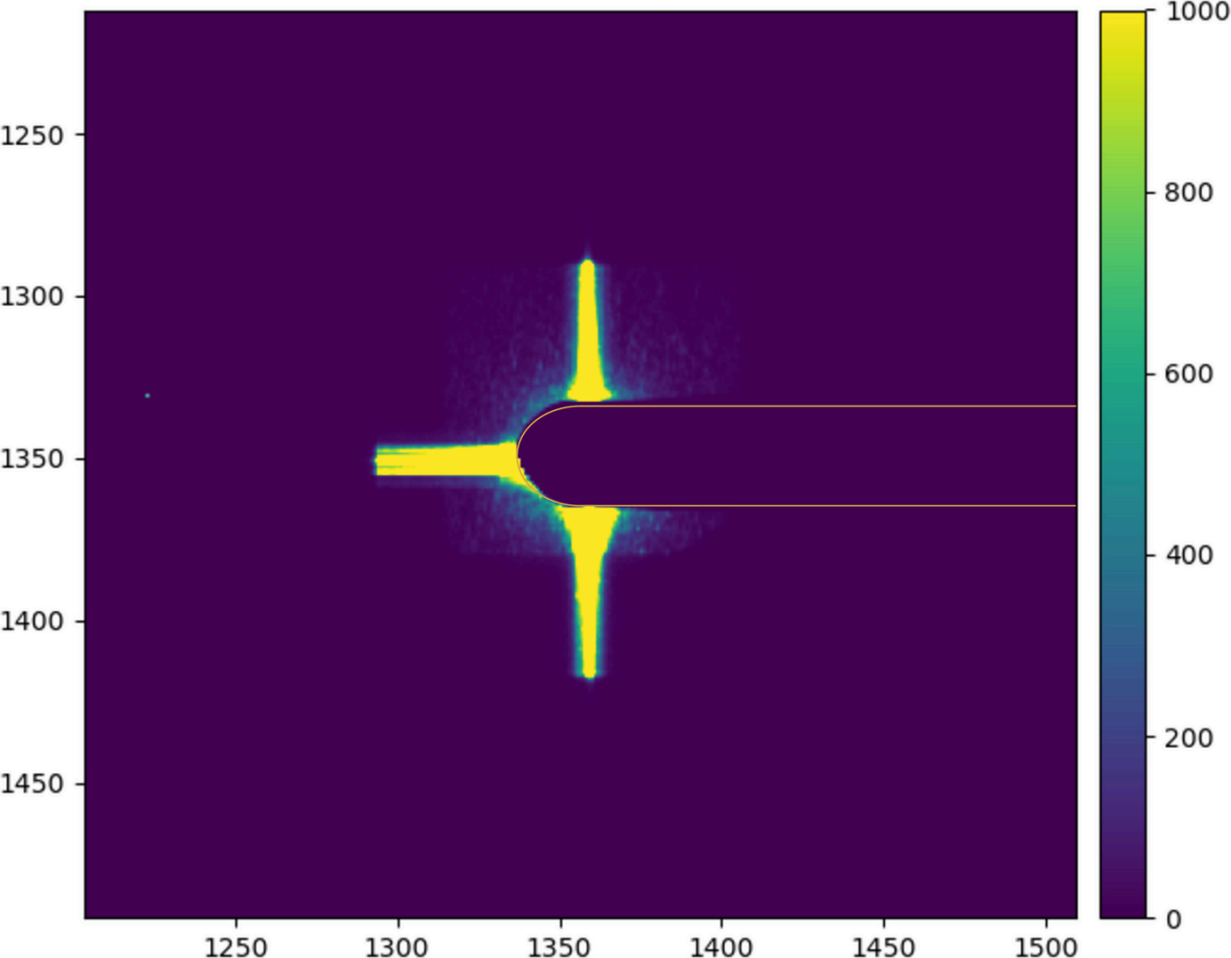
Background scattering measured with a pixelated detector positioned in forward scattering and 7 m from the sample. The data correspond to an integration time of 1 s exposure time (with a flux of ∼1.7 × 10^12^ photons s^−1^). Due to the DPU, no contributions are visible. Orange: the outline of the beamstop used to block the direct beam. The visible streaks (cross-like pattern) are due to diffraction from the polished slits.

It is important to stress that, at high scattering angles (>0.25°), these flares did not create any detectable effect on the data. Since forward scattering experiments of weakly scattering systems are performed regularly at ID10-COH, we opted to mount a 500 µm aperture as A_1_. The flares basically disappear resulting in a clean small angle scattering background as shown in Fig. 5 ▸. Note that the cross-like pattern visible in Fig. 5 ▸ is due to diffraction from the polished slit blades (Le Bolloc’h *et al.*, 2002 ▸).

During most experiments, the vacuum-compatible sample environments are used and are connected on the upstream side to the DPU via a flexible bellow, while on the downstream side the sample chamber is connected to the ‘detector tube’ via KF40 bellow. The ‘detector tube’ is 4–7 m long and has a diameter of 200 mm.

For replacing the sample, the gate valve is closed [see Fig. 4 ▸(*d*)] and the sample chamber vented. No intervention is needed on the beamline side. After having replaced the sample, the sample chamber is pumped down before the gate valve is opened again. The full procedure takes less than 4 min and is risk-free for the beamline vacuum thanks to the presence of the DPU.

**Figure 6:**
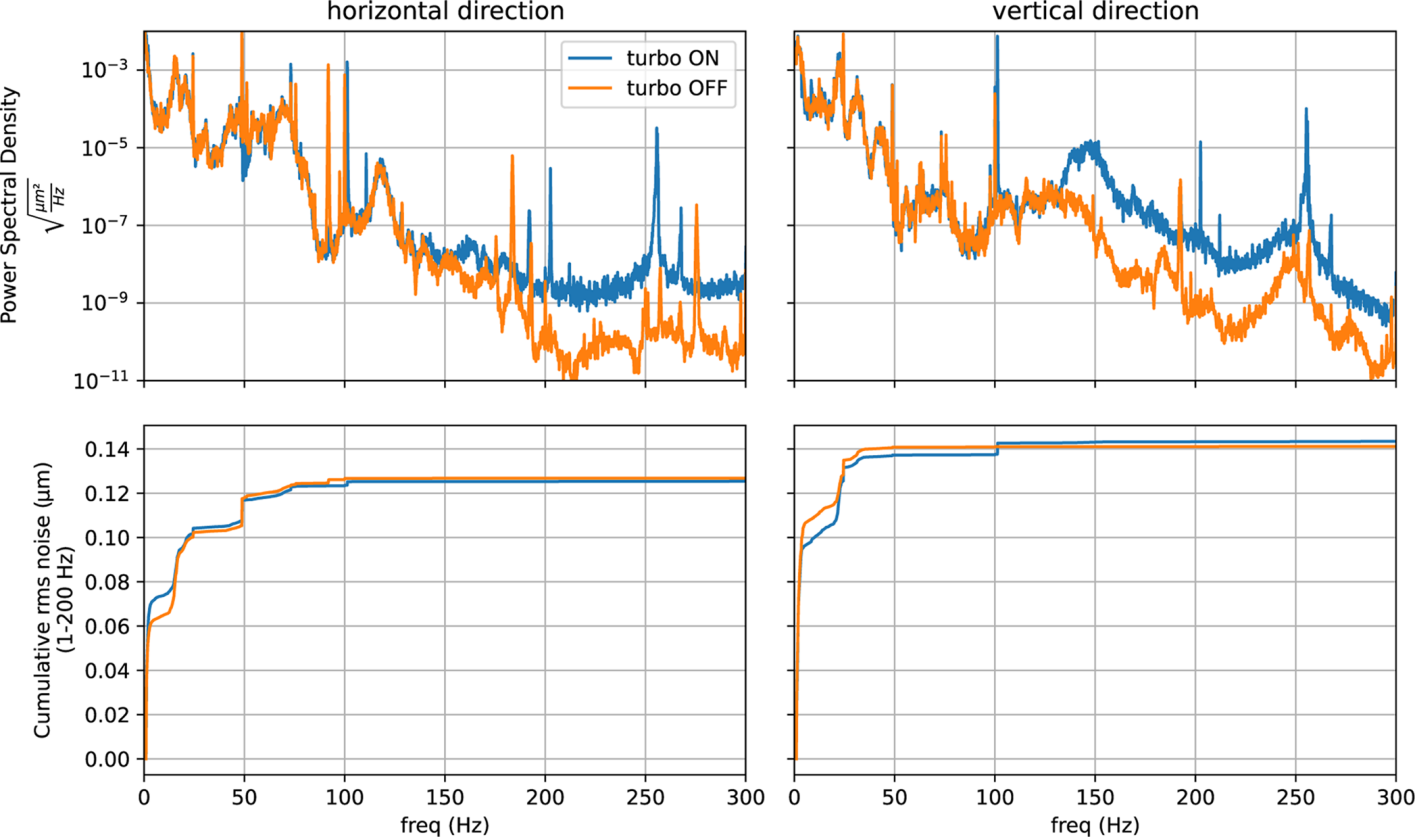
Power spectral density (top panels) and cumulative r.m.s. noise (lower panels) in the horizontal and vertical directions with the turbopump switched on and off. Data have been collected using 3D accelerometers with a bandwidth of 1–800 Hz. From the measured acceleration, the displacement power spectral density and cumulative r.m.s. noise have been calculated. Data have been collected with the turbopump on and off. Despite some increase noised that is observable at high frequencies (above 100 Hz), the associated displacement is relatively small as shown by the cumulative r.m.s. noise. We conclude that the effect of using the turbopump is negligible in our configuration.

Since the slits connected to the DPU are, for some experiments, used as beam defining slits, their positional stability is very important. Care was taken to measure the position stability (at rs2) using a high-sensitivy accelerometer with and without the turbopump switched on. Data, shown in Fig. 6 ▸, suggest that no significant loss of stability can be attributed to the turbopump operation.

## 5. Conclusions and outlook

The use of the DPU has greatly simplified the use of fully in-vacuum sample environments, limiting background scattering and background instabilities. Its operation has proven to be simple and reliable. Even when operated in air, the low power consumption of the turbopump (<20 W) results in a fan-free use in view of the modest temperature increase (a few degrees).

The modest cost (<15000 Euros including turbopump) and the flexibility of the design make this unit a good template that can be adapted – by changing the number of the stages and the size of the apertures and the pumps – to different endstations. Note the increased safety during beamline operation: thanks to the DPU, the beamline vacuum section is protected even in case of operator errors. Also note that the design is quite compact [368 mm versus 590 mm in Tamenori (2010 ▸) or 1500 mm in Madsen *et al.* (2013 ▸)] and able to sustain almost an 11 orders of magnitude reduction in pressure (from ambient to low 10^−8^ mbar).

At ID10-COH, more differential pumping based strategies will soon be exploited to remove the last two remaining windows: the diamond window that separates the beamline and the storage ring, and a Be window present at the entrance of the experimental hutch, thus allowing true windowless operation (from source to beamstop).

## Figures and Tables

**Table 1:** Calculated and measured pressures (during laboratory testing) Apertures were of the following diameters: A_1_ = 0.2 mm, A_2_ = 0.5 mm, A_3_ = 1 mm, A_4_ = 1 mm; 15 m^3^ h^−1^ primary pump with 2 m-long 10 mm-diameter rubber tube.

	*P*_1_ (mbar)	*P*_2_ (mbar)	*P*_3_ (mbar)	*P*_4_ (mbar)	Turbo power (W)
	Primary	Side port	Inlet	Ion pump	
Calculated	1.8	10^−2^	4.7 × 10^−6^	1.2 × 10^−8^	<40
Measured	2.0	2 × 10^−2^	4.0 × 10^−6^	–	33

**Table 2:** Calculated and measured pressures at ID10-COH Apertures using the following diameters were used: A_1_ = 0.5 mm, A_2_ = 0.5 mm, A_3_ = 1 mm; *P*_1_ pumped by 40 m^3^ h^−1^ primary pump via 4 m-long, 25 mm-diameter rubber tube. Turbopump fore-line pumped separately.

	*P*_1_(mbar)	*P*_2_ (mbar)	*P*_3_ (mbar)	*P*_4_ (mbar)	Turbo power (W)
	Primary	Side port	Inlet	Ion pump	
Calculated	3.6	2 × 10^−2^	9.4 × 10^−6^	2.4 × 10^−8^	–
Measured	4.2	1.8 × 10^−2^	7.1 × 10^−6^	–	18

## Footnotes

1 Flares were observed when the beam was focused to ∼2 m after the sample resulting in a beam size at the sample position of 25–30 µm. Note that in such configuration both rs1 and rs2 slits are used as cleanup slits. With the best settings (rs1 open to ∼130 µm and rs2 to ∼80 µm) notable flares were still visible.
